# Genetic Variations in the Kir6.2 Subunit *(KCNJ11)* of Pancreatic ATP-Sensitive Potassium Channel Gene Are Associated with Insulin Response to Glucose Loading and Early Onset of Type 2 Diabetes in Childhood and Adolescence in Taiwan

**DOI:** 10.1155/2014/983016

**Published:** 2014-09-21

**Authors:** Yi-Der Jiang, Lee-Ming Chuang, Dee Pei, Yann-Jinn Lee, Jun-Nan Wei, Fung-Chang Sung, Tien-Jyun Chang

**Affiliations:** ^1^Department of Internal Medicine, National Taiwan University Hospital, 7 Chung-Shan South Road, Taipei 10002, Taiwan; ^2^Graduate Institute of Preventive Medicine, School of Public Health, National Taiwan University, Taipei 10002, Taiwan; ^3^Division of Endocrinology and Metabolism, Department of Internal Medicine, Cardinal Tien Hospital, Xindian 23148, Taiwan; ^4^Department of Pediatrics, Mackay General Hospital, Taipei 10449, Taiwan; ^5^Chia Nan University of Pharmacy and Science, Tainan 71710, Taiwan; ^6^Institute of Environmental Health, College of Public Health, China Medical University, Taichung 40447, Taiwan

## Abstract

To investigate the role of E23K polymorphism of the *KCNJ11* gene on early onset of type 2 diabetes in school-aged children/adolescents in Taiwan, we recruited 38 subjects with type 2 diabetes (ages 18.6 ± 6.6 years; body mass index percentiles 83.3 ± 15.4) and 69 normal controls (ages 17.3 ± 3.8 years; body mass index percentiles 56.7 ± 29.0) from a national surveillance for childhood/adolescent diabetes in Taiwan. We searched for the E23K polymorphism of the *KCNJ11* gene. We found that type 2 diabetic subjects had higher carrier rate of E23K polymorphism of *KCNJ11* gene than control subjects (*P* = 0.044). After adjusting for age, gender, body mass index percentiles, and fasting plasma insulin, the E23K polymorphism contributed to an increased risk for type 2 diabetes (*P* = 0.047). K23-allele-containing genotypes conferring increased plasma insulin level during OGTT in normal subjects. However, the diabetic subjects with the K23-allele-containing genotypes had lower fasting plasma insulin levels after adjustment of age and BMI percentiles. In conclusion, the E23K variant of the *KCNJ11* gene conferred higher susceptibility to type 2 diabetes in children/adolescents. Furthermore, in normal glucose-tolerant children/adolescents, K23 allele carriers had a higher insulin response to oral glucose loading.

## 1. Introduction

Diabetes mellitus in children and adolescents has long been considered primarily type 1 diabetes. Although type 2 diabetes (T2D) is generally considered to be a disease of adults, the past 15–20 years have seen a dramatic increase in the prevalence of T2D in children and adolescents [1–9]. This increased prevalence of pediatric T2D suggests impending future morbidity from diabetic complications in a large number of relatively young adults.

In a nationwide surveillance program with mass urine screening in Taiwan [10, 11], the incidence of T2D is 6 times that of type 1 diabetes in recent years. The identified risk factors for T2D in youth are similar to those for adult type 2 diabetes with the most prominent risk of childhood obesity for T2D [11]. T2D is generally believed to be a polygenic disorder, with disease development being influenced by both hereditary and environmental factors [12]. Genetic factors are important in determining the children who become obese and also the obese children who develop T2D [13]. Support for the role of genetic factors comes from epidemiological evidence that T2D in youth is most common in individuals from racial groups with a high prevalence of diabetes and in individuals with a strong family history [14]. A search for the contribution of certain candidate genes in the early onset T2D is mandatory for further understanding of pathogenesis of T2D in childhood.

The pancreatic islet ATP-sensitive potassium channel complex (K_ATP_) plays a major role in glucose-stimulated insulin secretion, thus serving as a strong candidate for T2D. This channel is a heterooctameric complex composed of four sulfonylurea receptor (SUR1) subunits and four Kir6.2 subunits [15, 16]. Mutations in the SUR1 (*ABCC8*) and the Kir6.2 (*KCNJ11*) cause familial hyperinsulinemia in infancy [17], while some polymorphisms in these genes (exon 16-3t/c and exon 18 C/T of* ABCC8* and E23K of* KCNJ11*) have been reported to be associated with T2D in several populations at different degrees [18–23]. The K23 allele is associated with higher risk of T2D, providing an overall odds ratio (OR) of 1.23 [23] and 1.26 [21] in Caucasians and Asians, respectively. According to a recent systemic meta-analysis, the E23K polymorphism was significantly associated with increased T2D risk with per-allele odds ratio (OR) of 1.12. When stratified by ethnicity, significantly increased risks were found for the polymorphism in Caucasians and East Asians. However, no such associations were detected among Indian and other ethnic populations [24]. Normoglycemic lysine carriers are shown to consistently display a defect in insulin secretion [23, 25, 26]. Furthermore, the codon 23* KCNJ11* polymorphism is shown to be related to glucose intolerance in Caucasians and progression from glucose intolerance to T2D [27, 28].

Recent studies have provided evidence that the E23K variant alters channel function by inducing spontaneous overactivity of pancreatic *β*-cells, thus increasing the threshold of ATP concentration for insulin release [29, 30]. Therefore, in this study, we analyzed the E23K polymorphism of* KCNJ11* gene in a group of subjects with T2D and a group of controls identified in a nationwide surveillance program for diabetes in schoolchildren aged 6~18 in Taiwan. We demonstrated that the E23K polymorphism of* KCNJ11* gene increased susceptibility to T2D in childhood and adolescence.

## 2. Materials and Methods

### 2.1. Subjects

With a nationwide surveillance program for diabetes in Taiwanese school-aged children, 137 subjects were newly diagnosed with T2D [10, 11]. Only 38 newly diagnosed T2D subjects and 69 nondiabetic subjects from the northern part of Taiwan were recruited for genetic analysis. Body mass index (BMI) is a measure of body fat based on body height (BH) and body weight (BW) (BMI = BW (kg)/BH^2^ (m^2^)). After BMI is calculated for children and teens, the BMI number is plotted on the Centers for Disease Control and Prevention (CDC) BMI-for-age growth charts (for either girls or boys) to obtain a percentile ranking (http://www.cdc.gov/healthyweight/assessing/bmi/childrens_bmi/about_childrens_bmi.html, searched on 8.14.2014). A standard oral glucose tolerance test with 1.75 g glucose/kg of body weight or maximally with 75 g glucose was performed to classify the state of glucose tolerance, except for subjects diagnosed with a fasting plasma glucose level equal to or over 126 mg/dL. Informed consent was obtained from each participant and their parents of those under 18. This study was approved by the Institutional Review Boards.

### 2.2. Measurements of Metabolic Parameters

The fasting plasma glucose, serum insulin, cholesterol, and TG were measured according to the previous reports [31]. Insulin resistance index was calculated with homeostasis model assessment (HOMA-IR) as described previously [32]. The estimated *β*-cell function based on the HOMA-B was calculated based on the following formula: %B = 20 × fasting plasma insulin (FPI, *μ*U/mL)/(fasting plasma glucose (FPG, mM) − 3.5) [33]. Another set of estimates of *β*-cell function proposed by Stumvoll et al. was also calculated using the two formulae: 1st PH_s_ = 1283 + (1.829 × plasma insulin concentration at 30 min) − (138.7 × plasma glucose concentration at 30 min) + (3.772 × FPI) and 2nd PH_s_ = 287 + (0.4164 × plasma insulin concentration at 30 min) − (26.07 × plasma glucose concentration at 30 min) + (0.9226 × FPI). These estimations were based on plasma glucose concentrations in mmol/L and plasma insulin concentrations in pmol/L [34]. Insulinogenic index (30 minutes) was estimated as follows: (Ins 30 − Ins 0)/(Glu 30 − Glu 0) [35]. Area under curve (AUC) of glucose and insulin during OGTT was also calculated.

### 2.3. Genotyping for the Polymorphism of* KCNJ11*

The E23K polymorphism of* KCNJ11* was genotyped by PCR-restriction fragment length polymorphism (PCR-RFLP). PCR was performed with forward primer 5′-GACTCTGCAGTGAGGCCCTA-3′ and reverse primer 5′-ACGTTGCAGTTG CCTTTCTT-3′ starting with a denaturing step at 95°C for 3 min followed by 35 cycles of 95°C for 30 s, annealing at 60°C for 30 s, and elongation at 72°C for 30 s with a final elongation step at 72°C for 9 min. The PCR product was 209 bps, and it was digested with* Ban*II (New England Biolabs, Beverly, MA) and separated on 3% agarose gels. The substitution of G with A eliminated the* Ban*II site.

### 2.4. Statistical Analysis

Data were represented as mean ± SD. Due to relatively small sample size, EK/KK were grouped together for regression analyses. Fisher's exact test was used to detect the distribution difference between diabetic and nondiabetic groups. Logistic regression model was further performed to adjust demographic difference. Student's *t*-test was applied to compare the difference of various parameters between different genotypes or between normal control and diabetic subjects. MANOVA was applied to compare the difference of glucose and insulin levels during OGTT test between different genotypes. SAS program version 8.1 (SAS institute Inc., Cary, NC) was applied for statistical analyses. A value of *P* < 0.05 was considered statistically significant.

## 3. Results

### 3.1. Demographic and Metabolic Characteristic

The demographic and metabolic data of the study subjects are shown in Table 1. Obesity, dyslipidemia, higher fasting plasma insulin, higher insulin resistance, and worsened *β*-cell function were found in subjects with T2D in childhood and adolescence (Table 1).

### 3.2. Genotypes of* KCNJ11* Genes

As shown in Table 2, K-allele-containing genotypes were significantly higher in subjects with T2D as compared to those of control (84.2% versus 65.2%, *P* = 0.044) (Table 2). To further adjust for potential confounding variables, logistic regression analysis was performed (Table 3). After adjustment of age, sex, and BMI age- and sex-specific percentiles (model 1), we found that higher BMI percentiles is an independent risk factor of type 2 diabetes (odds ratio = 1.060, 95% CI: 1.027–1.094, and *P* < 0.001). If we adjust for age, sex, BMI age- and sex-specific percentiles, and fasting plasma insulin levels (model 2), the K-allele-containing genotype is an independent risk factor of type 2 diabetes (odds ratio = 4.105, 95% CI: 1.0008–16.831, and *P* = 0.047). The fasting plasma insulin levels and BMI age- and sex-specific percentiles are also independent risk factors for T2D (odds ratio = 1.066, 95% CI: 1.001–1.135, and *P* = 0.045 for fasting insulin; odds ratio = 1.047, 95% CI: 1.014–1.080 for BMI age- and sex-specific percentiles, and *P* = 0.004, resp.).

### 3.3. Effect of E23K Polymorphism of the* KCNJ11* in Normal Glucose-Tolerant Subjects

**Figure 1:**
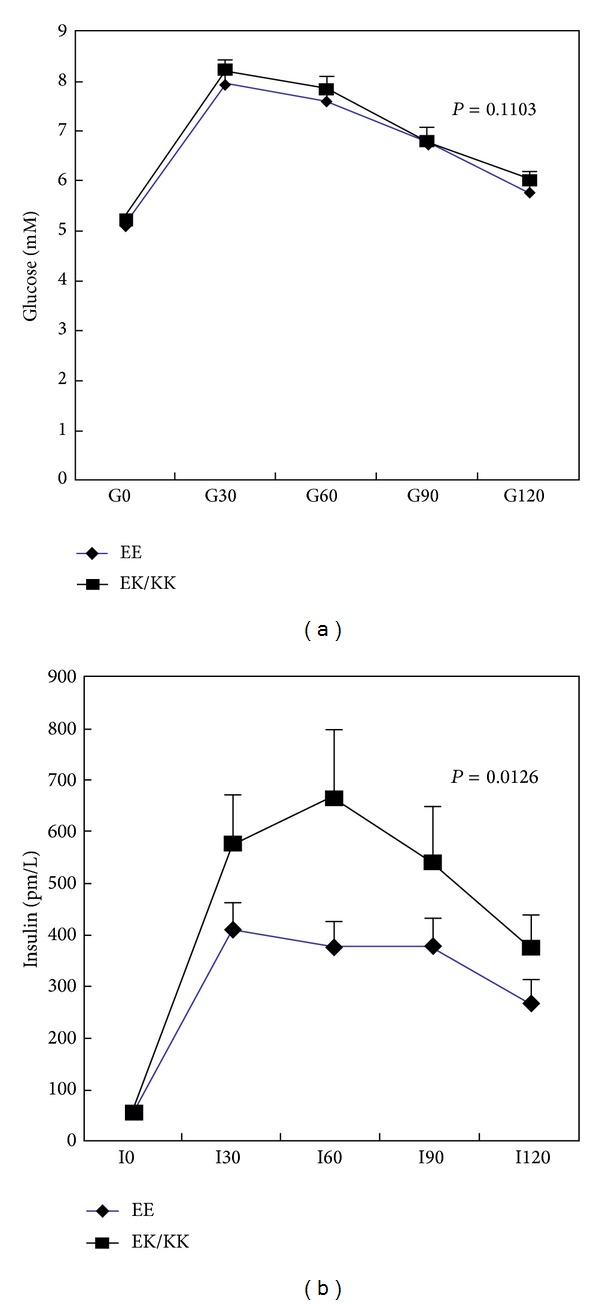
Glucose and insulin homeostasis during OGTT in the normal glucose-tolerant subjects according to genotypes of the* KCNJ11* gene. There was no significant difference in plasma glucose levels in subjects with EE genotype compared with those with K23-allele-containing genotypes (EK or KK) (a). Subjects with EK/KK genotypes tended to have a higher level of plasma insulin level during OGTT (b). ∗ indicates significant difference between the two groups.

To study the effect of genetic polymorphism of the E23K on insulin and glucose homeostasis during oral glucose tolerance test, we firstly compared those with EE genotype and the K23-allele-containing genotypes (EK or KK) in the normal glucose-tolerant subjects. There was no difference in the glucose levels during OGTT between subjects with different genotypes (Figure 1(a)). In contrast, subjects with EK/KK genotypes did have a significantly higher level of plasma insulin level at 60 min after glucose loading and higher AUC of insulin during OGTT (Figure 1(b)). However, this association of genotype of* KCNJ11* with plasma insulin levels at 60 min after glucose loading became insignificant after adjustment of age, sex, and BMI percentiles.

### 3.4. Effect of E23K Polymorphism of the* KCNJ11* on Clinical Phenotypes in Diabetic and Nondiabetic Subjects

To further search for the features of E23K variants on development of type 2 diabetes in children and adolescents, we then compared various metabolic parameters between those with EE genotype and the K23-allele-containing genotypes (EK or KK) in both diabetic and nondiabetic subjects (Table 4). In general, the fasting insulin levels were higher in diabetes group. However, the diabetic subjects with the K23-allele-containing genotypes had a borderline significantly lower level of fasting plasma insulin than the diabetic subjects without K23 allele. With adjustment for age and BMI age- and sex-specific percentiles, the diabetic subjects with K-allele-containing genotypes had significantly lower fasting plasma insulin levels (fasting plasma insulin as dependent variable: *β* for age: −2.958 ± 1.385, *P* = 0.043; *β* for BMI age- and sex-specific percentiles: 0.548 ± 0.238, *P* = 0.030; *β* for E/E or E/K + K/K genotype: −21.451 ± 10.312, *P* = 0.046). In contrast, there were no significant differences of these metabolic parameters between the K23-allele-containing and the EE genotype subjects in the nondiabetic group.

## 4. Discussion

In our present study, we found that a common polymorphism of E23K of the* KCNJ11* confers higher susceptibility to T2D in childhood and adolescence of the Han-Chinese in Taiwan.

Childhood obesity is the single most important risk factor for type 2 diabetes in our schoolchildren [10, 11] and the present study. In the present study, we found that even with adjustment for age, sex, BMI age- and sex-specific percentiles, and fasting plasma insulin level, the K-allele-containing genotypes (EK and KK) confer an independent risk, with a relative high odds ratio of 4.105, for T2D in youth. Until recently, the Pro12Ala polymorphism in the peroxisome proliferator-activated receptor gamma (*PPARG*) was one of the other few polymorphisms that demonstrated an alteration in type 2 diabetes susceptibility across different populations [36]. More recently, several genome-wide association studies (GWAS) independently confirmed the strong associations of SNP rs7903146 in the* TCF7L2* locus with type 2 diabetes [37–40]. Evidence accumulated so far suggests that the E23K polymorphism of the* KCNJ11* gene, which encodes the Kir6.2 subunit of the K_ATP_ channel, is a candidate gene for type 2 diabetes reported mostly from adults [24, 41]. In children, one recent study indicated that six single nucleotide polymorphisms, including an activating R201H mutation on* KCNJ11* gene, contribute to permanent neonatal diabetes [42]. Besides, several mutations on* KCNJ11* gene have been reported to cause permanent hyperinsulinemic hypoglycemia of infancy [43–45]. A recent study reported that E23K variant did not affect metabolic disorders in prepubertal children who is small for gestational age at birth [46]. On the other hand, the association of the E23K polymorphism with type 1 diabetes was not statistically significant in the evaluated Korean population [47]. To our knowledge, no study has been reported for the impact of E23K polymorphism of the* KCNJ11* gene on the early onset type 2 diabetes in children/adolescents. In consistence with previous studies in adult populations, we showed that school-aged children/adolescents with T2D in this study had higher E23K carrier rate of* KCNJ11* gene than normal subjects. According to Genetic Power Calculator (S. Purcell et al., 2003; http://pngu.mgh.harvard.edu/~purcell/gpc/), the estimated number of diabetic cases for 80% power will be 181 with *P* value less than 0.05. Though only 38 diabetic subjects were recruited in our study group, the E23K polymorphism still contributed to a significantly increased risk for type 2 diabetes independent of age, gender, BMI age- and sex-specific percentiles, and fasting plasma insulin level. Furthermore, from the meta-analysis of candidate-gene studies and GWAS for T2D in adults, the average odds ratio of each genetic variant to increased T2D risk is in the range from 1.10 to 1.37 [48]. According to a recent systemic meta-analysis, the E23K polymorphism was significantly associated with increased T2D risk with per-allele odds ratio (OR) of 1.12. However, in this study, the odds ratio of K-allele of* KCNJ11* to increased T2D risk in childhood and adolescence reached 4.105 after adjusting age, gender, BMI age- and sex-specific percentiles, and fasting plasma insulin levels. It inferred that the E23K polymorphism of* KCNJ11* contributed a much higher risk to T2D in children and adolescence than in adults.

How E23K variation leads to diabetes is not completely understood. In previous studies, it has been shown that insulin secretion is significantly reduced in both heterozygous (E/K) and homozygous (K/K) variants among the normal glucose-tolerant adults [49, 50]. In contrast, we found that those carrying K-allele exhibited a higher insulin response after oral glucose loading in the normal glucose-tolerant children (Figure 1(b)). In support of our findings, studies in the glucose-tolerant offspring of T2D patients carrying the E23K variants demonstrated significantly higher 2-hour insulin concentrations compared with those with control subjects [51]. Moreover, the E23K variant has been linked to an increase in BMI in the Danish population [50]. Taken together, these data including ours suggest that the higher response in insulin secretion to oral glucose loading might be due to the compensatory hypersecretion of insulin to maintain normal glucose homeostasis in the presence of insulin resistance. In spite of the small case number in this study, we found that there is a decline in fasting plasma insulin levels in diabetes subjects carrying K-alleles compared to those with homozygous EE genotype when adjusted for age, sex, and BMI age- and sex-specific percentiles. Whether the reduced fasting insulin levels observed in the diabetic children/adolescents with K23-allele-containing genotypes are due to inadequate compensation of *β*-cell failure is not known. Future longitudinal study will be required to establish the effect of E23K polymorphism in the* KCNJ11* gene on changes of body build, insulin resistance, and *β*-cell dysfunction during disease progression.

## 5. Conclusions

In conclusion, a common E23K variant of the* KCNJ11* gene conferred higher susceptibility to T2D in children/adolescents in Taiwan. Furthermore, in the normal glucose-tolerant children and adolescents, K23 allele carriers had a significantly higher insulin response to oral glucose loading, suggesting a compensatory insulin secretion in the presence of insulin resistance. However, the functional impact of the E23K polymorphism on progression of glucose intolerance and diabetes needs further investigation.

## Figures and Tables

**Table 1:** Clinical and metabolic features between normal controls and type 2 diabetic subjects in the present study.

	Non-DM (*n* = 69)	DM (*n* = 38)	*P* value∗
Age (years)	17.3 ± 3.8	18.6 ± 6.6	0.289
BMI percentiles	56.7 ± 29.0	83.8 ± 15.4	<0.001
Glucose (mmol/L)	5.19 ± 0.40	10.65 ± 4.05	<0.001
TCH (mmol/L)	4.05 ± 0.77	4.22 ± 1.02	0.4159
TG (mmol/L)	0.74 ± 0.26	1.17 ± 0.45	0.0005
HDL (mmol/L)	1.43 ± 0.39	1.20 ± 0.50	0.0320
Fasting insulin (pmol/L)	7.66 ± 4.47	17.99 ± 20.43	0.0043
HOMA-IR	1.78 ± 1.06	7.48 ± 8.09	<0.001
Log (HOMA-IR)	0.402 ± 0.624	1.449 ± 1.143	<0.001
HOMA-B	94.62 ± 58.17	94.13 ± 168.52	0.987
Log (HOMA-B)	4.372 ± 0.625	3.490 ± 1.458	0.001
Sex (M : F)	26 : 43	20 : 18	0.157^$^

*P value with Student's *t*-test.

^$^By chi-squared test.

BMI percentiles: body mass index age- and sex-specific percentiles; TCH: total cholesterol; TG: triglyceride; HDL: high density lipoprotein-cholesterol; HOMA-IR: homeostasis model assessment-insulin resistance; HOMA-B: homeostasis model assessment-*β* cell.

**Table 2:** Genotypic distribution of E23K polymorphism of the Kir6.2 between normal control and type 2 diabetes subjects.

	Non-DM (*n* = 69)	DM (*n* = 38)	*P* value∗	Odds ratio	95% confidence interval
Genotype∗					
EE, *n* (%)	24 (34.8%)	6 (15.8%)			
EK/KK, *n* (%)	45 (65.2%)	32 (84.2%)	0.044	2.84	1.04–7.75
Allele					
E-allele, *n* (%)	81 (58.7%)	38 (50.0%)			
K-allele, *n* (%)	57 (41.3%)	38 (50.0%)	0.251	1.42	0.81–2.50

*Chi-squared test.

**Table 3:** Logistic regression analysis with type 2 diabetic status as dependent variables, age, sex, BMI age- and sex-specific percentiles, and genotype of E23K polymorphism in Kir6.2 as independent variables.

Independent variables	Odds ratio	95% CI	*P* value
Model 1			
EK/KK versus EE	2.941	0.764–11.323	0.117
Age (every 1 year increment)	1.156	0.920–1.454	0.214
Sex (male = 1, female = 2)	0.672	0.224–2.019	0.479
BMI age- and sex-specific percentiles (every 1 percentile increment)	1.060	1.027–1.094	**<0.001**
Model 2			
EK/KK versus EE	4.105	1.0008–16.831	**0.047**
Age (every 1 year increment)	1.240	0.960–1.601	0.099
Sex (male = 1, female = 2)	0.826	0.252–2.711	0.752
BMI age- and sex-specific percentiles (every 1 percentile increment)	1.047	1.014–1.080	**0.004**
Fasting plasma insulin levels (every 1 pmol/L increment)	1.066	1.001–1.135	**0.045**

**Table 4:** Clinical and metabolic features between those with E/E genotype and K-containing allele among type 2 diabetic and nondiabetic subjects, respectively.

	Type 2 diabetic subjects			Nondiabetic subjects		
Genotypes	E/E (*n* = 6)	E/K + K/K (*n* = 32)	*P* value∗	E/E (*n* = 24)	E/K + K/K (*n* = 45)	*P* value∗
Sex (M : F)	4 : 2	16 : 16	0.663^$^	11 : 13	15 : 30	0.434^$^
Age (years)	21.5 ± 9.7	18.0 ± 5.8	0.242	17.2 ± 3.3	17.4 ± 4.1	0.861
BMI age- sex-specific percentiles	84.02 ± 22.07	83.73 ± 14.31	0.970	56.65 ± 25.63	56.79 ± 30.87	0.987
Glucose (mmol/L)	10.0 ± 3.8	10.8 ± 4.1	0.715	5.1 ± 0.4	5.2 ± 0.4	0.133
Fasting plasma insulin levels (pmol/L)	237.21 ± 230.48	112.18 ± 126.24	0.076	52.2 ± 31.1	56.5 ± 32.8	0.602
Ins-30′	—	—		414.67 ± 53.23	582.09 ± 94.71	0.222
Ins-60′	—	—		381.81 ± 51.51	671.57 ± 132.39	**0.046**
Ins-90′	—	—		379.09 ± 55.41	545.79 ± 107.51	0.280
Ins-120′	—	—		268.32 ± 48.95	375.18 ± 67.10	0.283
AUC-glucose	—	—		832.92 ± 21.83	855.72 ± 17.02	0.422
AUC-insulin				40074.46 ± 4153.74	60457.87 ± 10721.20	0.180
HOMA-B	127.0 ± 194.8	89.89 ± 168	0.685	97.69 ± 70.03	92.98 ± 51.55	0.751

*P value with Student's *t*-test.

^$^ *P* value with Fisher's exact test.
